# Doxycycline Alters Metabolism and Proliferation of Human Cell Lines

**DOI:** 10.1371/journal.pone.0064561

**Published:** 2013-05-31

**Authors:** Ethan Ahler, William J. Sullivan, Ashley Cass, Daniel Braas, Autumn G. York, Steven J. Bensinger, Thomas G. Graeber, Heather R. Christofk

**Affiliations:** 1 Department of Molecular and Medical Pharmacology, David Geffen School of Medicine, University of California Los Angeles, Los Angeles, California, United States of America; 2 Institute for Molecular Medicine, David Geffen School of Medicine, University of California Los Angeles, Los Angeles, California, United States of America; 3 Crump Institute for Molecular Imaging, David Geffen School of Medicine, University of California Los Angeles, Los Angeles, California, United States of America; 4 Jonsson Comprehensive Cancer Center, David Geffen School of Medicine, University of California Los Angeles, Los Angeles, California, United States of America; 5 Broad Stem Cell Research Center, David Geffen School of Medicine, University of California Los Angeles, Los Angeles, California, United States of America; University of Alabama at Birmingham, United States of America

## Abstract

The tetracycline antibiotics are widely used in biomedical research as mediators of inducible gene expression systems. Despite many known effects of tetracyclines on mammalian cells–including inhibition of the mitochondrial ribosome–there have been few reports on potential off-target effects at concentrations commonly used in inducible systems. Here, we report that in human cell lines, commonly used concentrations of doxycycline change gene expression patterns and concomitantly shift metabolism towards a more glycolytic phenotype, evidenced by increased lactate secretion and reduced oxygen consumption. We also show that these concentrations are sufficient to slow proliferation. These findings suggest that researchers using doxycycline in inducible expression systems should design appropriate controls to account for potential confounding effects of the drug on cellular metabolism.

## Introduction

The tetracycline family is a class of broad-spectrum antibiotics that have been used clinically since the mid-twentieth century. Since then, they have found application beyond their anti-microbial activity in both the clinic and biomedical research [1]–[3]. They are widely used in the latter context as mediators of inducible gene expression systems, but often with little discussion of or control for potential off-target effects they may have on mammalian cells. Because the tetracyclines have been shown to inhibit matrix metalloproteinases, retard proliferation, induce apoptosis, and impair mitochondrial function in various experimental settings, we were interested to determine whether these drugs can alter cellular metabolism at concentrations commonly used in inducible systems [4]–[12].

The canonical prokaryotic target of the tetracyclines is the bacterial ribosome, the inhibition of which blocks bacterial protein synthesis [1]. But there is significant evidence that tetracyclines can impair mitochondrial function in eukaryotic cells by inhibiting translation at the mitochondrial ribosome, an observation that has been explained by the origin of these organelles as endosymbiotic bacteria [8], [12]–[15]. Despite a reportedly weak interaction between the antibiotics and the mitochondrial ribosome, at high concentrations they have been shown to impair synthesis of proteins encoded in the mitochondrial genome–many of which are involved in oxidative metabolism–and promote a shift towards glycolysis [4].

In this study, we expanded upon these findings to determine potential confounding effects of the tetracyclines–particularly doxycycline (Dox), the predominantly used compound–at concentrations commonly employed in inducible gene expression systems: 100 ng/mL - 5 µg/mL. We found that these concentrations of drug can significantly alter the metabolic profile of the cell, as well as reduce the proliferative rate, though the effect size depends upon the particular cell line used. These data strongly suggest that researchers using Dox-inducible systems should carefully optimize experiments to minimize potentially confounding effects of the drug, and design additional controls as needed.

## Results

### Doxycycline Induces Metabolic Gene Expression Changes in Human Cells

**Figure 1:**
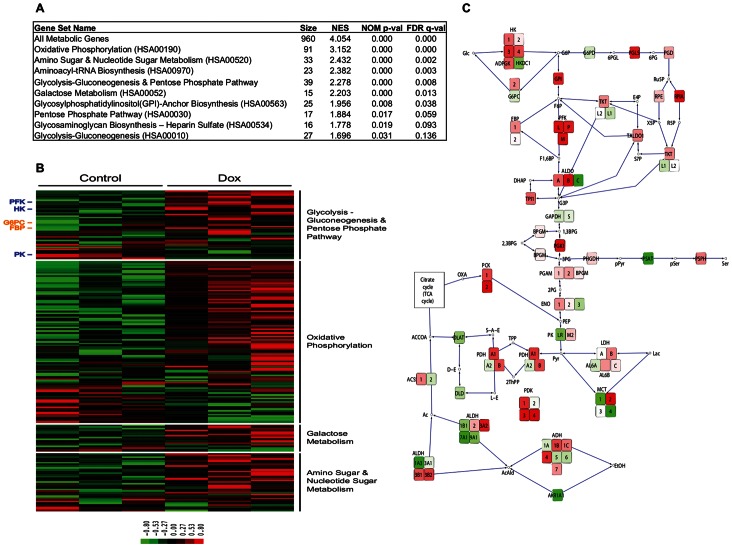
Doxycycline alters the metabolic gene expression profile of MCF12A cells. Treatment of MCF12A cells with Dox at a concentration of 1 µg/mL shows widespread changes in expression of metabolic genes. **A)** GSEA reveals the most significantly altered metabolic pathways, ranked by normalized enrichment score (NES), in Dox treatment compared to vehicle. KEGG pathway entries are denoted in parentheses where appropriate. Pathways without KEGG entries–All Metabolic Genes and Glycolysis-Gluconeogenesis & Pentose Phosphate–are artificial combinations of other pathways with redundant genes collapsed. All Metabolic Pathways includes all non-redundant genes from every KEGG pathway analyzed. **B)** This heat map highlights changes in the constituent genes of the oxidative phosphorylation and glycolysis/gluconeogenesis/pentose phosphate pathways upon treatment. Annotated genes include those encoding regulatory enzymes in glycolysis (phosphofructokinase (PFK), hexokinase (HK), pyruvate kinase (PK), shown in blue) and in gluconeogenesis (glucose-6-phosphatase (G6PC) and fructose-1,6-bisphosphatase (FBP), shown in orange). (**C)** Altered expression of regulatory enzymes in glycolysis and its proximal carbon shunts are shown schematically, with red indicating upregulation and green indicating downregulation.

To look in an unbiased way at the effects of Dox on cells in culture, we performed gene expression analysis on MCF12A cells–an untransformed breast epithelial line–treated with the drug at 1 µg/mL or with a vehicle control. Metabolic pathway enrichment analysis (using Gene Set Enrichment Analysis (GSEA)) revealed several pathways, including oxidative phosphorylation and glycolysis, to be significantly enriched in the Dox-treated cells (Figure 1A; for enrichment plots, see Figure S1). Many of the constituent genes in these pathways show a robust change in expression in response to treatment (Figure 1B; for annotated gene sets, see Figure S2), including key enzymes in glycolysis and its major carbon shunts (Figure 1C). These results demonstrate that Dox, at a concentration commonly used in inducible systems, can alter the metabolic gene expression profile of cells.

### Doxycycline Increases Glycolytic Metabolism in Multiple Human Cell Lines

**Figure 2:**
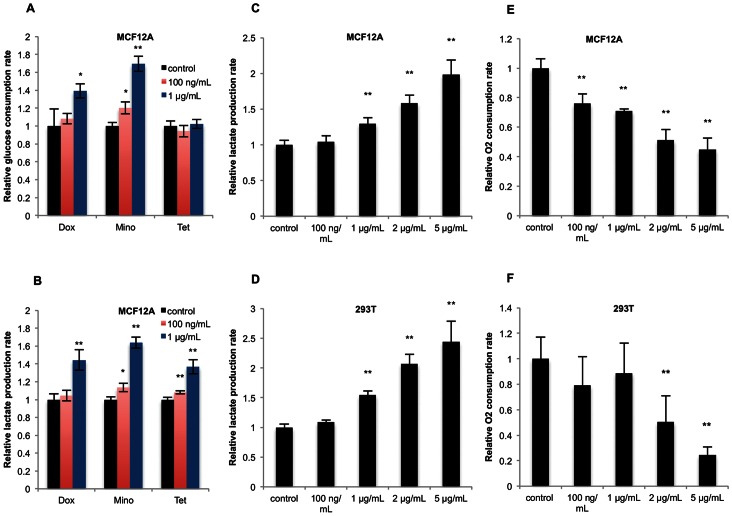
Tetracycline antibiotics affect glucose metabolism and oxygen consumption in a dose-dependent fashion. Effects of the tetracyclines on glycolytic flux were determined by measuring changes in **A)** glucose consumption and **B)** lactate production rates of MCF12A cells when treated with Dox, Mino, or Tet at 100 ng/mL or 1 µg/mL. A dose-response relationship was established between Dox and **C)** lactate production rate in MCF12A cells, **D)** lactate production rate in 293T cells, **E)** oxygen consumption rate in MCF12A cells, and **F)** oxygen consumption rate in 293T cells by treating with a range of drug concentrations. Error bars represent standard deviation from experimental triplicate measurements for all assays, except for oxygen consumption dose-response in 293T cells, which was performed with 8 replicates. * denotes a p-value ≤0.05, ** a p-value ≤0.01 from a two-tailed Student’s t-test.

Because treatment with Dox alters expression of genes involved in glycolysis and oxidative phosphorylation, we tested whether Dox treatment causes corresponding functional metabolic changes in MCF12A cells. As shown in Figure 2, glucose consumption (Figure 2A) and lactate production rates (Figure 2B) are elevated in MCF12A cells after 96 hours of treatment with 1 µg/mL Dox. The Dox analogs tetracycline (Tet) and minocycline (Mino) were also tested at 1 µg/mL. Both drugs induced increased lactate production rates (Figure 2B), and Mino concomitantly caused increased glucose uptake–though this latter phenotype is not observed with Tet (Figure 2A). Significant changes were also observed at 100 ng/mL with Mino and Tet, but the effect size is considerably smaller.

To determine whether the effect of Dox on cellular metabolism is dose-dependent, we measured lactate production rates (Figures 2C and 2D) and oxygen consumption rates (Figures 2E and 2F) of MCF12A and 293T cells over a range of Dox doses commonly used experimentally (100 ng/mL–5 µg/mL). We found that the amount of Dox used correlates with the shift towards a glycolytic phenotype, as shown by increased lactate production and decreased oxygen consumption. Higher doses of Dox (10 µg/mL) lead to cytotoxic effects (data not shown). In this range of concentrations, there appears to be a clear relationship between the Dox dose and the magnitude shift towards glycolysis and away from oxidative metabolism in MCF12A and 293T cells. These results suggest that the tetracycline family of antibiotics can promote glycolytic metabolism in human cell lines.

**Figure 3:**
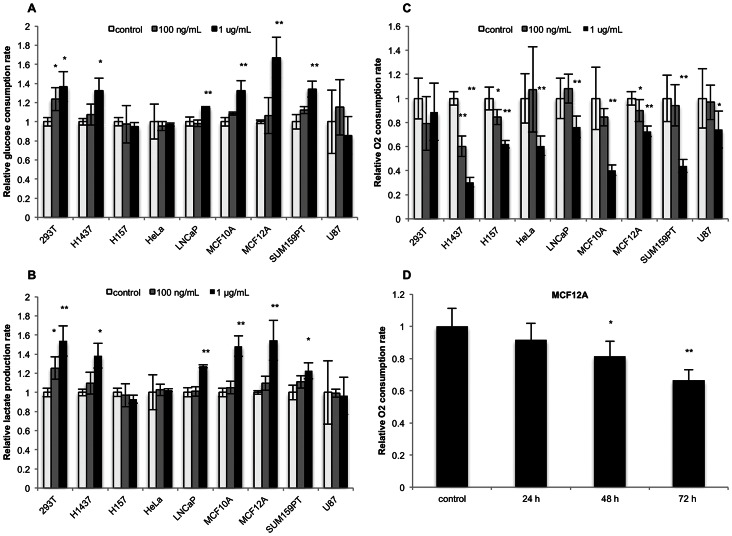
Doxycycline can alter both glycolytic and oxidative metabolism in a heterogeneous panel of human cell lines. Findings were extended to a panel of human cell lines, which were treated with Dox for 96 hours at 100 ng/mL, 1 µg/mL, or with vehicle control and then assayed for **A)** glucose consumption rate, **B)** lactate production rate, and **C)** basal oxygen consumption rate. **D)** To assess the temporality of this phenotype, MCF12A cells were treated with Dox at 1 µg/mL at shorter time points–24, 48, and 72 hours–and oxygen consumption rate was measured. Error bars represent standard deviation from experimental triplicate measurements in 3A and 3B; from 6 (100 ng/mL) or 7 (control, 1 µg/mL) replicates in 3C; and from 5 replicates in 3D. Oxygen consumption measurements for 293T cells from Figure 2F were repurposed for 3C. * denotes a p-value ≤0.05, ** a p-value ≤0.01 from a two-tailed Student’s t-test.

To assess whether the Dox-mediated metabolic changes are cell type-specific or broadly generalizable to human cell lines, we repeated the metabolic assays on 9 different human cell lines–both transformed and untransformed–and observed enhanced glycolysis in a majority of them (6/9, for both glucose consumption and lactate production rates) (Figures 3A and 3B). There is considerable evidence suggesting that the tetracyclines interact with the mitochondrial ribosome and inhibit translation of transcripts from the mitochondrial genome. Accordingly, we measured basal oxygen consumption rates in the cell line panel after 96 hours of Dox treatment (Figure 3C) [14]. In all cell lines but 293T, oxygen consumption was significantly impaired at 1 µg/mL; three lines showed significant defects at 100 ng/mL. The magnitude of the decrease varied among the cell lines, with H157 cells experiencing a 70% reduction in oxygen consumption, despite having unaffected glycolytic flux. To understand the temporality of this phenotype, we measured oxygen consumption after treating MCF12A cells with 1 µg/mL Dox for 24, 48, and 72 hours, which revealed a significant reduction in the oxygen consumption rate post 48 hours of treatment (Figure 3D). Together, these data demonstrate that Dox concentrations commonly used in genetic systems can substantially reprogram metabolism in human cell lines, and that the phenotype can vary significantly among cell lines.

### Doxycycline Reduces Proliferation of Multiple Human Cell Lines

**Figure 4:**
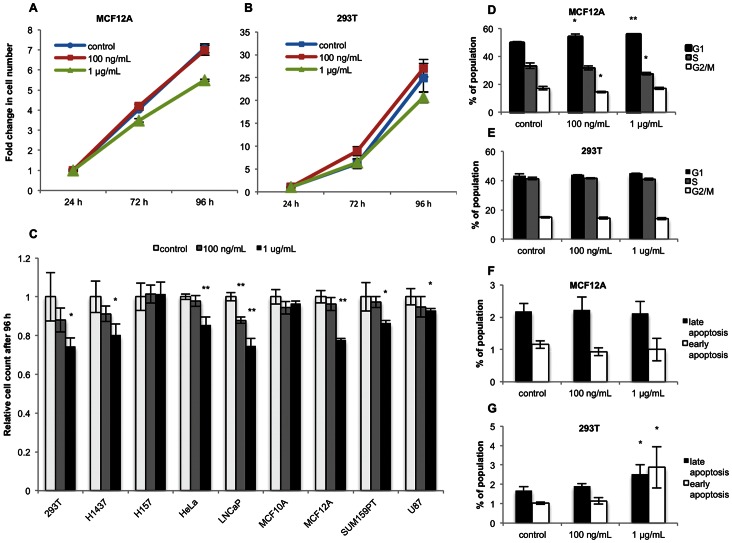
Doxycycline reduces proliferation of multiple human cell lines. Cell numbers were measured over 96 hours of Dox treatment. Shown here are representative growth curves for **A)** MCF12A and **B)** 293T cells, as well as **C)** the full panel of cell lines with cell counts after 96 hours of treatment normalized to vehicle control. To interrogate the reason for the proliferation defect, PI-based cell cycle analysis was performed on **D)** MCF12A and **E)** 293T cells, and Annexin V/PI viability analysis was performed on **F)** MCF12A and **G)** 293T cells. Both proliferation and flow cytometry-based assays were performed in triplicate, with error bars representing standard deviation. * denotes a p-value ≤0.05, ** a p-value ≤0.01 from a two-tailed Student’s t-test.

Previous studies have shown that Dox can impair cell proliferation *in vitro* [8]. Measuring cell number over a 96-hour time course, we found that at 1 µg/mL–but not 100 ng/mL–Dox treatment significantly reduces proliferation in most of the cell lines in our panel (7/9); only one cell line, the LNCaP prostate cancer line, experienced a proliferative defect at the lower concentration. Here, we show both representative growth curves for MCF12A and 293T cells (Figures 4A and 4B) and normalized values for the entire panel (Figure 4C). Interestingly, not all cell lines that experience substantial Dox-induced metabolic shifts have simultaneously impaired proliferative capacity–most clearly seen in MCF10A cells (Figures 3A–3C; Figure 4C)–suggesting potentially independent or decoupled mechanisms of action.

To determine what might cause reduced proliferation, cell cycle analysis was performed on MCF12A and 293T cells, both of which experience metabolic and proliferative changes with Dox treatment. Consistent with a previous report of tetracycline-induced G_1_ arrest, propidium iodide (PI) staining showed modest enrichment of cells in this phase of the cell cycle in MCF12A cells (Figure 4D), but not in 293T cells (Figure 4E) [5]. Because apoptosis has also been described in cells treated with tetracyclines, Annexin V/PI co-staining was performed on the same cells (Figures 4F and 4G) [5], [8]. At the concentrations used here, Dox significantly increases apoptotic cell death in 293T cells (Figure 4G), but not in MCF12A cells (Figure 4F). Since the Dox-induced effects on cell cycle and apoptosis vary between the cell lines tested, the mechanistic link between Dox treatment and the observed modestly impaired proliferation remains elusive.

## Discussion

We show here that Dox, as well as other members of the tetracycline family, can cause substantial changes in cellular metabolism and impair proliferative capacity of human cell lines. Importantly, these phenotypes are induced at concentrations commonly used in inducible gene expression systems, which indicates a strong possibility of confounding effects in these experiments. Our findings are surprising given the presumed inactivity of these drugs in eukaryotic cells, and particularly relevant because metabolism and proliferation are fundamental properties of the cell–inextricable from many other phenotypes. While inducible gene expression systems are a powerful tool for probing gene function, it is advisable that researchers carefully optimize experiments in order to minimize the concentration of Dox and the concomitant confounding phenotype. Additional controls can also be designed so that the effects of the mediating drug are disentangled from that of the gene of interest.

Because we did not investigate the mechanism of action, we cannot fully explain how Dox causes the observed phenotypes. But the evidence of interactions between the drug and the mitochondria are compelling, and may contribute to our observations. Since subunits of electron transport chain complexes are encoded in the mitochondrial genome, a potential stoichiometric imbalance caused by decreased translation of these genes could lead to reduced capacity for oxidative metabolism and a compensatory shift towards glycolysis [12], [14], [15]. While further investigation is needed to refine our understanding of the effects of the drug on human cells, these results highlight important confounding phenotypes of Dox treatment and underscore the importance of rigorous experimental design when using this compound in inducible expression systems.

## Materials and Methods

### Cell Culture

U87 astrocytoma cells were a gift from Dr. Paul Mischel (UCSD), lung cancer cell lines H157 and H1437 from Dr. Steven Dubinett (UCLA), and LNCaP prostate cancer and HeLa cervical cancer cell lines from Dr. Steven Bensinger (UCLA), and all other cell lines from Dr. Frank McCormick (UCSF); all cell lines have been well characterized [16]–[20]. MCF12A and MCF10A cells were cultured in DMEM/F12 (1∶1) containing 5% horse serum, 1% penicillin/streptomycin, 10 µg/mL insulin (Invitrogen), 0.5 µg/mL hydrocortisone (Sigma), 20 ng/mL EGF (Peprotech), and 10 µg/mL cholera toxin (Sigma). HeLa and U87 cells were cultured in DMEM containing 10% FBS and 1% penicillin/streptomycin. H1437, H157, and LNCaP cells were cultured in RPMI containing 10% FBS and 1% penicillin/streptomycin. SUM159PT cells were cultured in F12 containing 5% FBS, 1% penicllin/streptomycin, 5 µg/mL insulin (Invitrogen), 1 µg/mL hydrocortisone (Sigma), and 0.1% gentamicin (Sigma). Doxycycline, tetracycline, and minocycline (Sigma) were dissolved in water. Media containing the drug was refreshed every 48 hours, as appropriate.

### Metabolic Pathway Enrichment Analysis

RNA extraction was performed using RNeasy (Qiagen) according to manufacturer’s guidelines. Samples were analyzed using the Affymetrix Genechip U133 Plus 2 array by the UCLA Clinical Microarray Core Laboratory. Resulting data are available through NCBI’s Gene Expression Omnibus (GEO Series accession number GSE45029). To identify metabolic pathways associated with Dox treatment, we used the GSEA algorithm and metabolic pathway annotation defined by the Kyoto Encyclopedia of Genes and Genomes (KEGG) (release #58). We collapsed gene expression probes based on enzyme activity (Enzyme Commission [EC] numbers) rather than on gene identity to avoid unequal representation of equivalent enzymatic function within pathways, thus emphasizing potential flux through the network. The metric used for gene ranking was the signal to noise ratio (SNR) between the Dox treatment and control. The probesets were collapsed by average SNR for GSEA, and by maximum absolute SNR for each enzyme in the heat maps. Probeset annotation was based on UniGene build #230 and UniGene identifiers were mapped to each EC using the gene names provided by KEGG.

### Media Metabolite Measurement

Media was collected from culture plates and analyzed for glucose and lactate concentrations using the Bioanalyzer 4 (Nova Biomedical). Cells were treated with Dox or vehicle control for 96 hours. Fresh media was added to plates between 20 and 24 hours before sample collection. Values were normalized to both cell number and time interval. Some statistical variability was observed among experiments, particularly with respect to glucose, because the effect size measured at these concentrations of Dox is small compared to the error of the assay. Lactate is a more consistently sensitive readout of glycolytic activity.

### Oxygen Consumption Measurement

Basal oxygen consumption rates were measured using the XF24 Extracellular Flux Analyzer (Seahorse Bioscience). Cells were treated with Dox or vehicle control for 96 hours, unless otherwise indicated. 18 hours prior to assay, cells were seeded at 50,000–80,000 cells per well (optimized for each cell line) in 24-well proprietary assay plates with between 3 and 8 replicates per treatment group. Oxygen consumption values were normalized to total protein using BCA Protein Assay Kit (Pierce). Because plating density around the oxygen sensors affects readings, there is considerable well-to-well variability. Because cell morphology can alter the distribution of cells in the well, different cell lines are measured with varying sensitivity.

### Proliferation Assay

Cells were seeded in 6 well plates at 50,000 cells per well and treated with Dox (100 ng/mL, 1 µg/mL, or vehicle control). Cells were counted 24, 72, and 96 hours after plating using a particle counter (Beckman Coulter).

### Cell Cycle Analysis

Cell cycle analysis was performed using propidium iodide (PI) staining. In brief, cells were trypsinized and resuspended in hypotonic DNA staining buffer (3.5 mM sodium citrate, 0.1 g/L propidium iodide, 0.3% Triton X-100, 20 mg/L Ribonuclease A) at a concentration of 10^6^ cells/mL and incubated for 20 minutes at room temperature in the dark. Analysis was performed using the BD FACSDiva software on an LSR II flow cytometer (BD Biosciences). PI fluorescence was recorded in the PE channel. For cell cycle analysis, flow cytometry data was imported and analyzed using the ModFit LT software.

### Viability Analysis

Cell viability was measured using the FITC Annexin V Apoptosis Detection Kit II (BD Pharmingen) according to the manufacturer’s protocol. Cells were incubated with Annexin V and PI for 15 min and then analyzed by flow cytometry as previously described. Annexin V fluorescence was recorded on the FITC channel and PI on the PE channel. Data was analyzed using FlowJo. Cells in early apoptosis were indicated by Annexin V positive, PI negative staining (lower right quadrant) while cells in late apoptosis were marked by Annexin V/PI double positive staining (upper right quadrant).

## Supporting Information

Figure S1 Metabolic gene sets are enriched upon treatment with Dox. Enrichment plots for the top ranked KEGG-defined pathways in MCF12A cells treated with Dox at 1 µg/mL, compared to vehicle control.
(TIF)

Figure S2 **Dox treatment alters expression of genes involved in major central carbon metabolism pathways.** Heat maps highlight changes in expression of genes in **A)** the Glycolysis-Gluconeogenesis & Pentose Phosphate Pathway (artificial combination of KEGG pathways) and in **B)** KEGG-defined Oxidative Phosphorylation after Dox treatment at 1 µg/mL in MCF12A cells, compared to vehicle control. Entries are ranked by SNR and are collapsed by enzyme function; the constituent gene with the maximum absolute SNR is shown in the heat map. Regulated enzymes in glycolysis are shown in blue and those in gluconeogenesis are shown in orange.
(PDF)
