# Supplementary figures and images for: Doxycycline Alters Metabolism and Proliferation of Human Cell Lines
Source: PLoS One. 2013 May 31;8(5):e64561. doi: 10.1371/journal.pone.0064561 (PMC3669316; doi:10.1371/journal.pone.0064561)

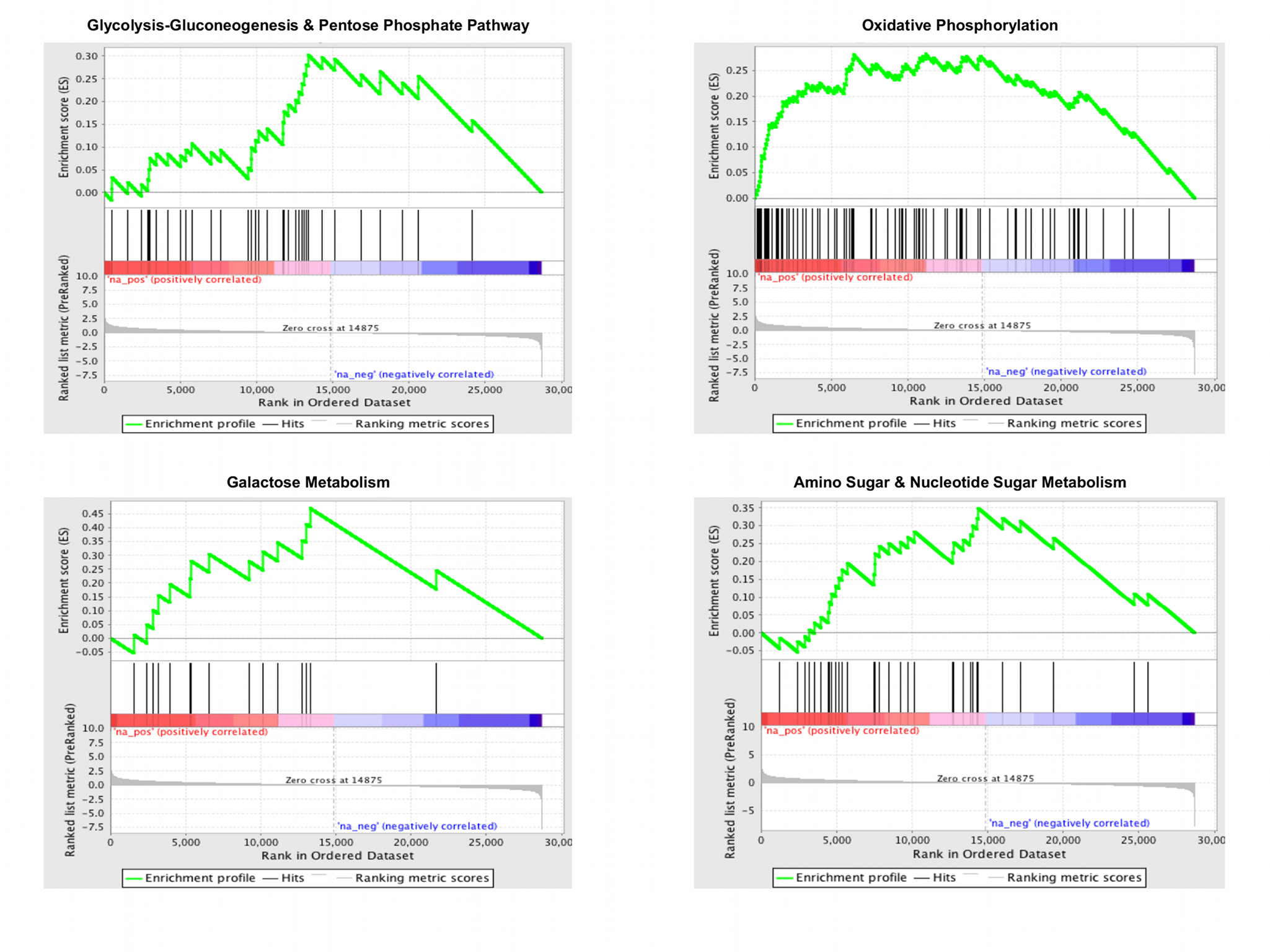

Supplement: Figure S1 — Metabolic gene sets are enriched upon treatment with Dox. Enrichment plots for the top ranked KEGG-defined pathways in MCF12A cells treated with Dox at 1 µg/mL, compared to vehicle control. (TIF) [file pone.0064561.s001.tif]

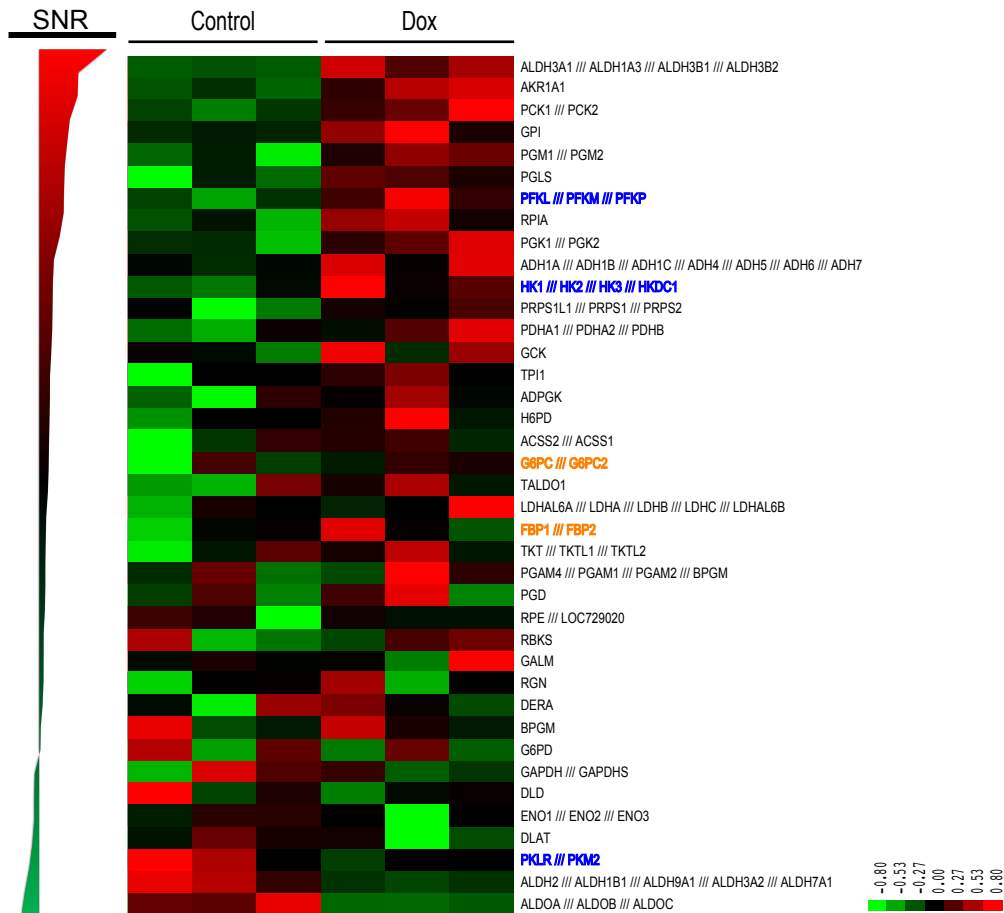

SNR

Control

Dox

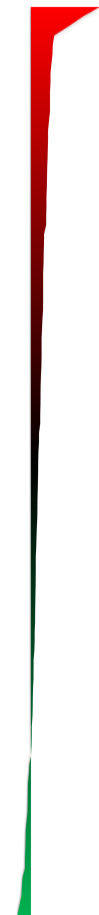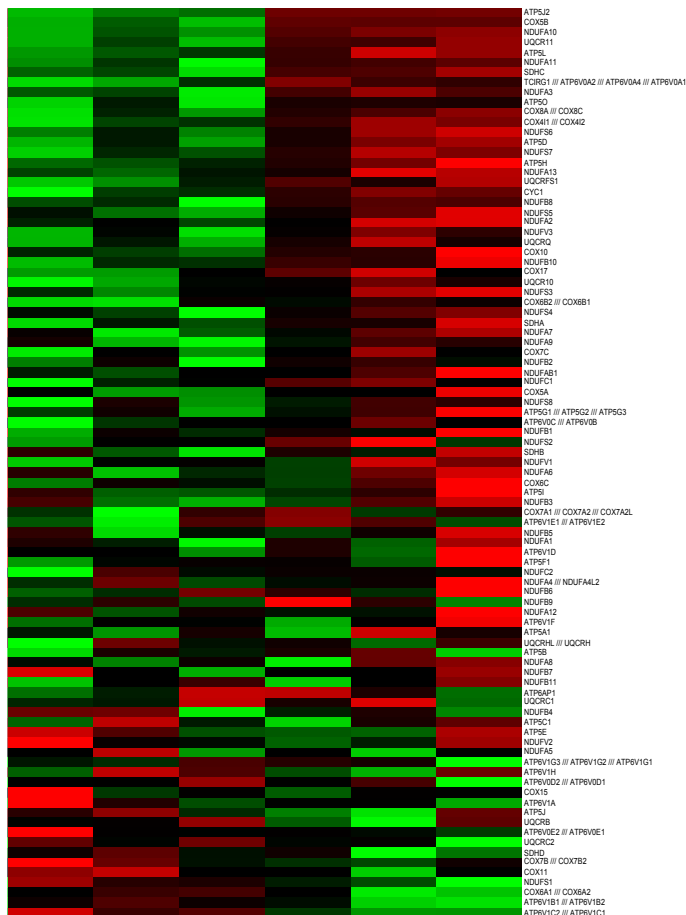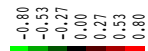

Supplement: Figure S2 — Dox treatment alters expression of genes involved in major central carbon metabolism pathways. Heat maps highlight changes in expression of genes in A) the Glycolysis-Gluconeogenesis & Pentose Phosphate Pathway (artificial combination of KEGG pathways) and in B) KEGG-defined Oxidative Phosphorylation after Dox treatment at 1 µg/mL in MCF12A cells, compared to vehicle control. Entries are ranked by SNR and are collapsed by enzyme function; the constituent gene with the maximum absolute SNR is shown in the heat map. Regulated enzymes in glycolysis are shown in blue and those in gluconeogenesis are shown in orange. (PDF) [file pone.0064561.s002.pdf]
